# Peer relationships as a moderator of the effect of grit on hope in a serial mediation model through hope and sports participation motivation predicting sports engagement among Chinese university students

**DOI:** 10.3389/fpsyg.2026.1790362

**Published:** 2026-03-04

**Authors:** Xiaoneng Wu

**Affiliations:** Department of Physical Education, Yangzhou Polytechnic University, Yangzhou City, Jiangsu Province, China

**Keywords:** Chinese college student, grit, hope, moderated mediation effect, peer relationships, sports engagement, sports participation motivation

## Abstract

This study explored the potential associations among grit, hope, sports participation motivation, and sports engagement among Chinese university students. Additionally, it examined whether peer relationship might play a moderating role in the indirect pathways between grit and sports engagement. Participants were selected using multistage stratified sampling combined with convenience sampling, resulting in a final sample of 509 college students enrolled in physical education or sports-related programs. Data were analyzed using IBM SPSS Statistics 25.0, AMOS 23.0, and the SPSS PROCESS macro 4.2. The analytical procedures included frequency analyses, reliability testing, exploratory factor analysis, confirmatory factor analysis, measurement model evaluation, and moderated mediation analysis using PROCESS Model 83. The results revealed two major findings. First, hope, peer relationship, sports participation motivation, and sports engagement were all significantly and positively intercorrelated. In contrast, grit was significantly negatively correlated with peer relationship but positively correlated with hope, sports participation motivation, and sports engagement. Second, peer relationships demonstrated a significant conditional indirect effect in the pathway from grit to sports engagement via hope and participation motivation. Specifically, peer relationships suppressed the positive effect of grit on hope and weakened the indirect pathway leading to engagement. These findings contribute to the theoretical advancement of peer relationship research by highlighting its potential to attenuate, rather than enhance, the motivational processes.

## 1 Introduction

Grit refers to sustained effort and consistent passion toward long-term goals (Duckworth et al., 2007), and has been recognized as a key psychological characteristic predicting various outcomes among university students, including academic achievement, career-related behaviors, and sports participation. In the context of sports, grit has been identified as an important personal resource that promotes persistence, engagement, and participation motivation. However, the translation of grit into actual behavioral outcomes cannot be fully understood without considering the broader social context in which individuals operate.

Hope, defined as a motivational resource encompassing pathway thinking and agency for goal attainment (Snyder, 2002), interacts closely with grit. Previous studies have shown that individuals with higher levels of grit tend to exhibit stronger expectations for goal achievement and more effective pathway planning, which in turn enhance their levels of hope. Elevated hope subsequently facilitates participation motivation and engagement in sports activities. Thus, the pathway from grit → hope → participation motivation → sports engagement has been consistently supported across cultural contexts.

Nevertheless, this pathway does not necessarily function uniformly across all environments. Peer relationships, in particular, represent a critical social factor influencing university students’ psychological adjustment and sports participation, and may serve as a potential moderator of the association between grit and hope. Prior research has predominantly emphasized the positive role of peer relationships, suggesting that emotional support, social bonding, and a sense of belonging provided by peers enhance individuals’ motivation and positive psychological functioning (Wentzel, 2017). From this perspective, peer relationships have generally been conceptualized as a positive moderator.

However, peer relationships do not always exert beneficial effects. Some studies have demonstrated that peer dynamics involving social comparison, peer pressure, or interpersonal conflict can undermine individuals’ psychological resources and motivation (Prinstein and Dodge, 2008; Rose and Asher, 2017). These findings indicate that peer relationships may function as sources of stress rather than support, potentially suppressing internal motivation and positive affect during goal pursuit. In the context of Chinese university students, cultural collectivism, heightened sensitivity to peer evaluation, and strong social comparison norms may further amplify the possibility that peer relationships operate as a detrimental rather than supportive factor.

Despite these insights, existing literature has overwhelmingly focused on the enhancing role of peer relationships, leaving the possibility that peer relationships may weaken the association between grit and hope largely unexplored. This gap suggests that even students with high levels of grit may fail to fully develop hope when embedded in negative or pressuring peer environments, ultimately weakening the pathway leading to sports engagement. Addressing this overlooked perspective offers meaningful theoretical and practical implications for understanding sports participation among Chinese university students.

Accordingly, the present study aims to explore the potential pathways linking grit to sports engagement through hope and sports participation motivation among Chinese university students. It further investigates whether peer relationship may play a moderating role within this process. In particular, this study considers the possibility that peer relationships may function as a suppressing moderator that attenuates the association between grit and hope, thereby tentatively addressing a gap in the existing literature.

To achieve these objectives, the study addresses the following research questions:

1. What are the correlations among the major variables (grit, hope, sports participation motivation, sports engagement, and peer relationships)?
2. Do peer relationships moderate the mediating effects of hope and participation motivation in the relationship between grit and sports engagement?

## 2 Theoretical background

### 2.1 Relationship between grit and sports engagement

Grit is widely conceptualized as a personality trait reflecting sustained perseverance and consistent passion toward long-term goals (Duckworth et al., 2007). Individuals high in grit maintain effort despite challenges and remain committed to their objectives over extended periods. Recent sport psychology literature further emphasizes grit as a dynamic psychological resource that supports athletes’ resilience and long-term development in competitive environments (Lee and Oh, 2025; Mendizabal, 2024). Sports engagement, on the other hand, refers to athletes’ behavioral, emotional, and cognitive involvement in sports-related activities. It encompasses active participation in training, psychological investment in performance, and sustained commitment to the sport. Engagement is shaped not only by internal motivation but also by contextual and psychological factors that influence athletes’ willingness to invest effort (Larkin et al., 2023). In team sports such as sports, engagement is often conceptualized as a multidimensional construct reflecting both observable behaviors and underlying motivational states.

A growing body of empirical research has demonstrated a positive association between grit and sports-related engagement. First, gritty athletes tend to accumulate more deliberate practice hours and achieve higher performance levels, indicating that grit facilitates sustained behavioral engagement in sports. Larkin et al. (2023) found that grit significantly predicted skill development among elite youth soccer players beyond the effects of deliberate practice alone, suggesting that perseverance plays a unique role in maintaining long-term involvement. Second, grit has been shown to enhance psychological well-being among collegiate athletes, which in turn supports their engagement in sport. A recent study by Kovács (2025) reported that grit positively predicted autonomy, environmental mastery, and personal growth—psychological resources known to promote sustained participation and emotional investment in athletic activities. Third, gritty athletes demonstrate more adaptive responses to setbacks, such as injuries or performance failures. Qualitative findings indicate that individuals high in grit interpret obstacles as temporary challenges rather than reasons to disengage, thereby maintaining their commitment to sports participation (Cormier et al., 2025).

These empirical findings align with major motivational theories. Social Cognitive Theory (Bandura, 1986) posits that personal characteristics such as perseverance influence behavioral outcomes through self-regulatory mechanisms, explaining why gritty individuals remain engaged in sports despite difficulties. Self-Determination Theory (Deci and Ryan, 2000) further suggests that grit enhances perceived competence and autonomy, which strengthens intrinsic motivation and supports sustained engagement. Together, these theoretical perspectives and empirical results indicate that grit plays a central role in promoting sports engagement by fostering persistence, psychological resilience, and long-term motivational investment.

### 2.2 Dual mediating roles of hope and sports participation motivation

Hope is conceptualized as a cognitive–motivational construct composed of agency thinking and pathway thinking, which together enable individuals to pursue goals effectively (Snyder, 2002). Hope reflects one’s perceived capacity to generate routes toward desired goals and sustain the motivation to follow those routes. Recent sport psychology research further defines hope as a psychological resource that enhances athletes’ confidence in achieving performance outcomes and facilitates persistence in physical activity contexts (Blythe et al., 2025). Sports participation motivation refers to the internal and external motives that drive individuals to engage in sports activities. It encompasses intrinsic motives such as enjoyment and competence, as well as extrinsic motives such as social recognition or fitness goals. Contemporary studies describe participation motivation as a multidimensional construct that predicts sustained involvement and commitment in sport (Tao and Yu, 2025).

A substantial body of research supports the mediating role of hope in the relationship between grit and sport-related outcomes. Grit has been shown to positively predict hope, as individuals with higher perseverance and long-term goal orientation tend to exhibit stronger agency and pathway thinking. Longakit et al. (2025) demonstrated that both components of grit significantly predicted dispositional hope among student-athletes. Similarly, Blythe et al. (2025) found that hope-agency was strongly associated with long-term goal pursuit and physical activity engagement, suggesting that gritty individuals are more likely to develop higher levels of hope. In turn, hope has been identified as a significant predictor of sport engagement. Blythe et al. (2025) reported that higher hope-agency predicted vigorous physical activity and greater goal attainment, while Longakit et al. (2025) found that hope significantly predicted sports motivation, a known antecedent of sport engagement. These findings indicate that hope mediates the relationship between grit and sports engagement by enhancing athletes’ motivational and cognitive resources.

Sports participation motivation also functions as a mediator linking grit to sports engagement. Grit has been shown to enhance participation motivation, as gritty individuals are more likely to maintain high levels of intrinsic and extrinsic motivation toward sport. Longakit et al. (2025) found that grit was a strong predictor of student-athletes’ motivation toward sports, while Kovács (2025) reported that athletes with higher grit demonstrated stronger sport orientation and persistence—key components of participation motivation. Participation motivation, in turn, is a well-established predictor of sport engagement. Tao and Yu (2025) demonstrated that exercise motivation significantly predicted sport participation and commitment among college students, and Larkin et al. (2023) found that motivated athletes accumulated more deliberate practice hours and showed higher engagement in sports-related activities. These findings support the mediating role of participation motivation in the grit–engagement relationship.

Furthermore, evidence suggests that hope enhances participation motivation, supporting a sequential dual mediation pathway. Longakit et al. (2025) reported that hope significantly predicted sports motivation, indicating that hopeful athletes are more motivated to participate in sport. Blythe et al. (2025) similarly found that hope-agency predicted exercise goal attainment, which is closely related to participation motivation. These findings support a dual mediation model in which grit increases hope, which then enhances participation motivation, ultimately leading to higher sports engagement.

Taken together, the literature provides strong support for the dual mediating roles of hope and sports participation motivation. Grounded in Hope Theory (Snyder, 2002) and Self-Determination Theory (Deci and Ryan, 2000), this framework highlights the importance of cognitive–motivational processes in explaining how grit influences sports engagement among university students.

### 2.3 Moderating role of peer relationships

Peer relationships constitute a critical social context influencing university students’ psychological development, motivation, and sport-related behaviors. In sport settings, peer relationships are commonly defined as the quality of interpersonal interactions, emotional support, acceptance, and social connectedness experienced among teammates or peers (Wentzel, 2017). High-quality peer relationships provide athletes with encouragement, belongingness, and positive feedback, which can enhance motivation and engagement. Conversely, negative peer dynamics—such as conflict, exclusion, or social comparison—can undermine psychological well-being and reduce motivation (Prinstein and Dodge, 2008). Thus, peer relationships function as a multidimensional social factor capable of exerting both facilitative and detrimental influences on athletes’ cognitive and motivational processes.

From a theoretical standpoint, Social Cognitive Theory (Bandura, 1986) posits that personal factors, environmental influences, and behaviors interact reciprocally. Within this framework, peer relationships serve as an environmental factor that can strengthen or weaken the translation of personal traits (e.g., grit) into cognitive–motivational outcomes (e.g., hope, participation motivation). Similarly, Self-Determination Theory (Deci and Ryan, 2000) emphasizes the importance of relatedness as a basic psychological need; supportive peer environments enhance intrinsic motivation, whereas negative peer interactions can frustrate this need and diminish motivation. These theoretical perspectives suggest that peer relationships may moderate the pathways through which grit influences hope, participation motivation, and ultimately sports engagement.

Empirical evidence supports the moderating role of peer relationships in sport and academic contexts. Positive peer relationship has been shown to enhance motivation, emotional well-being, and engagement. For example, Wentzel (2017) found that supportive peer interactions promoted academic motivation and adaptive functioning, highlighting the motivational benefits of positive peer environments. In sport settings, peer relationship has been linked to increased enjoyment, persistence, and engagement, suggesting that peers can reinforce athletes’ motivational processes (Smith et al., 2016). These findings imply that when peer relationships are positive, gritty individuals may experience stronger hope and higher participation motivation, thereby increasing sports engagement.

However, research also indicates that peer relationships can exert negative or suppressive effects. Prinstein and Dodge (2008) demonstrated that peer conflict, pressure, and negative social comparison can undermine adolescents’ psychological adjustment and motivation. Rose and Asher (2017) similarly reported that peer difficulties, such as exclusion or relational stress, negatively affected youths’ emotional functioning and goal pursuit. In sport contexts, negative peer dynamics have been associated with reduced motivation, lower enjoyment, and disengagement (Smith et al., 2016). These findings suggest that when peer relationships are poor, the positive effects of grit on hope and participation motivation may be weakened, thereby diminishing sports engagement.

Taken together, the literature indicates that peer relationships may function as a moderating factor that either strengthens or suppresses the indirect effects of grit on sports engagement through hope and participation motivation. Positive peer environments may amplify the motivational benefits of grit, whereas negative peer dynamics may inhibit the development of hope and reduce participation motivation. This dual potential underscores the importance of examining peer relationships as a contextual moderator within the broader motivational framework of sports engagement among university students.

## 3 Research methods

### 3.1 Research model

**Figure 1:**
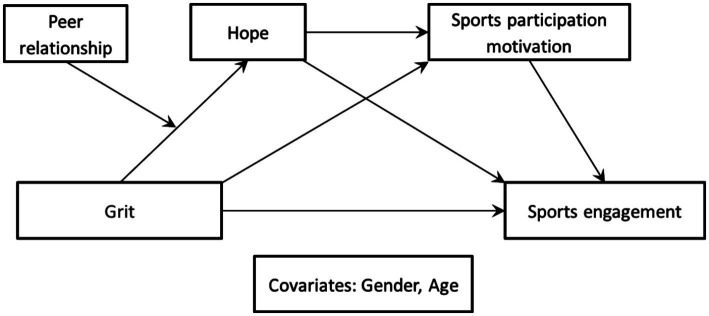
Research model.

The research model was developed based on prior empirical and theoretical studies to examine whether peer relationship moderates the sequential associations among grit, hope, sports participation motivation, and sports engagement. To assess this moderated mediation pattern, Model 83 of the SPSS PROCESS macro (Hayes, 2017) was employed. Grit was specified as the independent variable, sports engagement as the outcome variable, and hope and sports participation motivation as sequential mediators. Peer relationship was included as a moderator of the association between grit and hope. Gender and age were statistically controlled to account for potential confounding variables. The conceptual model is illustrated in Figure 1.

### 3.2 Participants and data collection method

To enhance geographic diversity, a multistage sampling strategy was employed. Five provinces representing eastern, western, southern, northern, and central China were selected, followed by the random selection of one city per province. Within each city, one higher education institution offering physical education or sports-related programs was purposively chosen to ensure ecological relevance to organized sport participation contexts.

A total of 625 students (125 per institution) were invited to participate. Institutional-level recruitment used convenience sampling, targeting students enrolled in sports classes or actively participating in sports clubs. Of the 625 invited students, 38 were automatically excluded prior to survey access through an online eligibility filter (not enrolled in sports classes or sports clubs). The remaining 587 eligible students accessed the survey, of whom 78 discontinued before completion. A total of 509 participants completed the questionnaire and were included in the final analysis, yielding an overall completion rate of 81.4%.

The survey platform required responses to all items, resulting in no missing data. No duplicate entries were detected, and no cases were excluded based on response time or quality-control criteria. Accordingly, all 509 completed questionnaires constituted the final analytic sample.

Participants provided electronic informed consent prior to survey access and were informed of their right to withdraw at any time. The study received ethical approval from the Institutional Review Board of Hanseo University (Approval No. HS25-12-01).

The final sample (*N* = 509) comprised 72.5% male and 27.5% female students, with a mean age of 18.9 years. First-year students accounted for 60.9% of the sample.

### 3.3 Research tools

### 3.4 Grit

Grit was measured using the Short Grit Scale (Grit-S) developed by Duckworth and Quinn (2009), which comprises two theoretically derived subdimensions: Consistency of Interest and Perseverance of Effort. Each subscale includes four items, resulting in a total of eight items. Of the eight items, four (Items 1–4) were reverse-coded prior to composite score computation. Higher composite scores indicate greater levels of grit. Participants responded using a 5-point Likert scale ranging from 1 (strongly disagree) to 5 (strongly agree).

To examine construct validity, exploratory factor analysis (EFA) and confirmatory factor analysis (CFA) were conducted. Principal axis factoring with Varimax rotation was employed for the EFA. The Kaiser–Meyer–Olkin value (0.806) and Bartlett’s test of sphericity (*χ*^2^ = 2474.356, *p* < 0.001) indicated adequate sampling adequacy. The EFA yielded a clear two-factor solution consistent with the original conceptualization of grit. All items loaded above 0.50 on their respective factors, and no problematic cross-loadings were observed (primary–secondary loading differences ≥0.15).

A subsequent CFA confirmed the two-factor structure. All standardized factor loadings exceeded 0.50, and the model demonstrated acceptable fit indices: *χ*^2^(4) = 15.469, *p* < 0.001; *χ*^2^/*df* = 3.867; GFI = 0.993; TLI = 0.967; CFI = 0.995; RMSEA = 0.075. The correlation between Perseverance of Effort and Consistency of Interest was moderate to substantial (*r* = 0.641), indicating meaningful relatedness while supporting the distinguishability of the two subdimensions.

Given this empirical structure, grit was retained as a two-factor construct in the measurement model. However, consistent with prior research that has operationalized grit as a general psychological strength using composite scores (e.g., Duckworth et al., 2007; Duckworth and Quinn, 2009; Credé et al., 2017), and in line with the study’s focus on overall perseverance-related functioning, a composite grit score was used in subsequent structural analyses. This approach preserves the multidimensional measurement structure while enabling parsimonious modeling of overall grit.

Convergent validity and reliability were evaluated based on the validated measurement structure. The average variance extracted (AVE) was 0.661, and composite reliability (CR) was 0.978. Internal consistency reliability was satisfactory (McDonald’s *ω* = 0.978; Cronbach’s *α* = 0.873), exceeding commonly recommended thresholds.

#### 3.4.1 Peer relationship

Peer relationship was measured using the Peer Relationship Scale originally developed by Asher (1978). The scale consists of 16 items rated on a 5-point Likert scale (1 = strongly disagree to 5 = strongly agree), designed to assess the perceived quality of peer interactions in school contexts. Example items include “It’s easy for me to make new friends at school” and “I cannot find anyone to talk to” (reverse-coded). Of the 16 items, 10 items (Items 2, 4, 6, 8, 9, 11, 12, 13, 14, and 16) were reverse-coded prior to computing the average score. Higher composite scores indicate higher peer relationships.

To evaluate the construct validity of the scale, both exploratory factor analysis (EFA) and confirmatory factor analysis (CFA) were conducted. For the EFA, principal axis factoring was employed as the extraction method, and Varimax rotation was applied to facilitate interpretability. The number of factors was determined using the eigenvalue-greater-than-one criterion, and only items with factor loadings of 0.50 or higher were retained. The results demonstrated excellent sampling adequacy, as indicated by a Kaiser–Meyer–Olkin (KMO) value of 0.935 and a significant Bartlett’s test of sphericity (*χ*^2^ = 5297.726, *p* < 0.001). Two distinct factors were extracted, with all items showing strong loadings above 0.60 on their respective factors. No problematic cross-loadings were observed; the difference in factor loadings between primary and secondary factors exceeded 0.15 for all items, supporting a clear factor structure.

Based on the EFA results, a CFA was subsequently conducted. The refined model demonstrated acceptable fit indices: *χ*^2^(84) = 308.601, *p* < 0.001; *χ*^2^/*df* = 3.674; GFI = 0.932; TLI = 0.949; CFI = 0.965; RMSEA = 0.073. Although peer relationship has been conceptualized as comprising two subdimensions, the present study operationalized it as a unidimensional construct. This decision was supported by the coherent factor structure observed in EFA and CFA, the strong factor loadings, and the high reliability indices, indicating substantial general factor saturation. Given that the research objective focused on overall perceived peer relationship rather than facet-level distinctions, the use of a global composite score was considered theoretically and psychometrically appropriate. Accordingly, convergent validity and reliability were evaluated based on the single-factor model. Convergent validity was supported by an average variance extracted (AVE) of 0.616 and a composite reliability (CR) of 0.997. Internal consistency was excellent (*ω* = 0.997; *α* = 0.910). Although the very high CR and ω values indicate strong factor coherence, coefficients exceeding 0.95 may also suggest substantial inter-item homogeneity. Therefore, these values were interpreted as reflecting strong general factor saturation while acknowledging the possibility of partial item redundancy.

Reverse-coded items (Items 2, 4, 6, 8, 9, 11, 12, 13, 14, and 16) were recoded prior to composite score computation and all subsequent analyses. Scoring consistency was verified by examining item–total correlations, factor loading directions, and the signs of correlations with key variables. These checks confirmed that higher scores consistently reflected more positive peer relationships across all analyses, and that scoring direction and association patterns were reported consistently across tables, results, and discussion sections.

#### 3.4.2 Hope

Hope was assessed using the hope scale developed by Snyder et al. (1991), which theoretically comprises two subdimensions: agency thinking and pathways thinking. Each subdimension consists of four items, resulting in a total of eight items. Participants responded on a 5-point Likert scale ranging from 1 (strongly disagree) to 5 (strongly agree), with higher scores indicating greater levels of hope.

To examine construct validity, exploratory factor analysis (EFA) and confirmatory factor analysis (CFA) were conducted. Principal axis factoring was used for the EFA. Contrary to the original two-factor conceptualization, the EFA in the present sample yielded a clear one-factor solution. The first factor had an eigenvalue substantially greater than 1, while subsequent factors fell below the threshold. The scree plot indicated a sharp inflection after the first factor. The single factor accounted for a substantial proportion of total variance, and all items loaded strongly (≥ 0.60), supporting empirical unidimensionality in this sample.

Based on the EFA results, a CFA was conducted to test a one-factor model of hope with eight observed indicators (Items 1–8). The initial model demonstrated acceptable but improvable fit. Examination of modification indices indicated substantial localized strain between specific item pairs within the same theoretical subdimensions. Accordingly, residual covariances were freed between Item 5 and Item 6, and between Item 7 and Item 8, as these items belong to the same subdimensions (agency or pathways) and share highly similar wording and conceptual content. These theoretically justified modifications resulted in improved model fit. The final model demonstrated excellent fit: *χ*^2^(15) = 34.066, *p* < 0.01; *χ*^2^/*df* = 2.271; GFI = 0.983; TLI = 0.987; CFI = 0.993; RMSEA = 0.050. All standardized factor loadings exceeded 0.50.

Although the Hope Scale has been traditionally conceptualized as comprising two related subdimensions (agency and pathways), the empirical evidence from the present study supported a dominant general factor. In addition to the unidimensional structure identified in the EFA, the correlation between the two theoretically derived factors was high (*r* = 0.824), indicating substantial overlap between agency and pathways thinking. Such a strong association suggests that the two components may reflect a common underlying construct of hope in this sample. Given this high inter-factor correlation and the strong general factor saturation observed in both EFA and CFA, modeling hope as a single latent construct was considered empirically justified and theoretically appropriate, particularly because the present study focused on overall levels of hope rather than differential effects of its subcomponents.

Convergent validity and reliability were evaluated based on the final one-factor CFA model. The average variance extracted (AVE) was 0.590, and composite reliability (CR) was 0.996. Internal consistency was high (McDonald’s *ω* = 0.996; Cronbach’s *α* = 0.918). Although reliability coefficients exceeding 0.95 may suggest substantial inter-item homogeneity, these values were interpreted as reflecting strong general factor coherence while acknowledging the possibility of content overlap across items.

#### 3.4.3 Sports participation motivation

Sports participation motivation was assessed using the scale adapted by Li (2016), grounded in self-determination theory (SDT). The instrument originally comprises six subdimensions—intrinsic motivation, integrated regulation, identified regulation, introjected regulation, external regulation, and amotivation—each measured by three items, yielding a total of 18 items. Participants responded on a 7-point Likert scale ranging from 1 (strongly disagree) to 7 (strongly agree), with higher scores indicating stronger motivation to participate in sports.

To examine construct validity, exploratory factor analysis (EFA) and confirmatory factor analysis (CFA) were conducted. Principal axis factoring with Varimax rotation was used for the EFA. The Kaiser–Meyer–Olkin value (0.967) and Bartlett’s test of sphericity (*χ*^2^ = 11016.93, *p* < 0.001) indicated excellent sampling adequacy. Contrary to the originally proposed six-factor structure, the EFA yielded a two-factor solution. Items reflecting relatively autonomous forms of motivation (e.g., intrinsic and identified regulation) loaded primarily on one factor, whereas items reflecting more controlled forms of regulation and amotivation loaded on the second factor. All factor loadings exceeded 0.50, and no problematic cross-loadings were observed (primary–secondary loading differences ≥ 0.15). The correlation between the two extracted factors was substantial (*r* = 0.718), indicating strong conceptual relatedness while suggesting partial distinguishability.

Based on the EFA results, a CFA was conducted. The two-factor model demonstrated acceptable model fit, and the correlation between the latent factors remained high. Given this substantial inter-factor correlation and the strong general factor saturation observed across items, an overarching motivational construct was considered plausible. Because the present study focused on overall engagement in sports participation rather than differential effects of autonomous versus controlled motivation, and because preliminary analyses indicated comparable structural patterns when using the two factors separately, sports participation motivation was operationalized as a single composite construct for parsimony and consistency in the unit of analysis across study variables.

The single-factor CFA model demonstrated acceptable fit: *χ*^2^(92) = 373.912, *p* < 0.001; *χ*^2^/df = 4.064; GFI = 0.927; TLI = 0.964; CFI = 0.978; RMSEA = 0.078. All standardized factor loadings exceeded 0.50. Convergent validity and reliability were evaluated based on this final one-factor model. The average variance extracted (AVE) was 0.782, and composite reliability (CR) was 0.997. Internal consistency was excellent (McDonald’s *ω* = 0.997; Cronbach’s *α* = 0.974). Although reliability coefficients above 0.95 may indicate substantial inter-item homogeneity, these values were interpreted as reflecting strong general motivational coherence while acknowledging potential conceptual overlap among regulation types.

#### 3.4.4 Sports engagement

Sports engagement was measured using the Athlete Engagement Scale (AES), originally developed by Lonsdale et al. (2007) and subsequently applied in a sports context by Cui (2022). The AES was theoretically conceptualized as comprising four related subdimensions: vitality, dedication, enthusiasm, and confidence. Each subdimension is represented by four items, resulting in a total of 16 items. Participants responded using a 5-point Likert scale, with higher scores indicating greater engagement in sports activities.

To examine construct validity, exploratory factor analysis (EFA) and confirmatory factor analysis (CFA) were conducted. Principal axis factoring was used for the EFA. The Kaiser–Meyer–Olkin measure indicated excellent sampling adequacy (KMO = 0.970), and Bartlett’s test of sphericity was significant (*χ*^2^ = 8863.861, *p* < 0.001), supporting the suitability of the data for factor analysis.

The EFA yielded a clear one-factor solution. The first factor had an eigenvalue of 12.267, whereas all subsequent eigenvalues were below 1.0. The extracted factor accounted for 75.17% of the total variance, indicating a strong dominant general factor underlying the 16 items. All items demonstrated substantial factor loadings (≥ 0.70), and no problematic cross-loadings were observed. Although this structure diverges from the original four-factor conceptualization, the results indicate strong empirical unidimensionality in the present sample, suggesting that vitality, dedication, enthusiasm, and confidence were highly integrated aspects of a single engagement construct.

Based on the EFA findings, a CFA was conducted to test the one-factor model. All standardized factor loadings exceeded 0.50, and the model demonstrated acceptable fit: *χ*^2^(75) = 261.767, *p* < 0.001; *χ*^2^/*df* = 3.49; GFI = 0.941; TLI = 0.972; CFI = 0.982; RMSEA = 0.070. Given the dominant general factor observed in the EFA and the satisfactory CFA fit indices, sports engagement was modeled as a single latent construct in subsequent analyses.

Convergent validity and reliability were evaluated based on the final one-factor CFA model. The average variance extracted (AVE) was 0.746, and composite reliability (CR) was 0.999. Internal consistency was exceptionally high (McDonald’s *ω* = 0.999; Cronbach’s *α* = 0.979). Although reliability coefficients approaching unity may reflect substantial inter-item homogeneity or potential content overlap, they are also consistent with the strong general factor saturation observed in the factor analysis. These values were therefore interpreted cautiously as evidence of a highly coherent engagement construct in this sample.

### 3.5 Common method Bias test

To assess the potential threat of common method bias (CMB), both procedural and statistical remedies were implemented. Procedurally, the study ensured respondent anonymity and varied the order of items across constructs to mitigate evaluation apprehension and reduce consistency motives—standard practices known to minimize the likelihood of bias.

To statistically address the potential influence of common method variance, a latent method factor model was tested by linking an unmeasured method factor to all observed indicators. This model showed slightly better fit indices (*χ*^2^/*df* = 5.073, CFI = 0.794, TLI = 0.779, RMSEA = 0.090) than the baseline measurement model (*χ*^2^/*df* = 6.487, CFI = 0.713, TLI = 0.702, RMSEA = 0.104).

While these changes indicate a potential presence of method effects, the overall model fit remained suboptimal, and the observed improvements in fit (ΔCFI < 0.10; ΔRMSEA < 0.02) did not exceed commonly accepted thresholds for meaningful change (Cheung and Rensvold, 2002). Therefore, any inferences about method variance should be made cautiously and considered preliminary.

Given these findings, caution is warranted in concluding that CMB poses no threat. Instead of relying solely on statistical controls, greater emphasis is placed on the procedural strategies adopted in the research design, which likely played a more critical role in mitigating method bias. Nevertheless, the presence of some residual method variance cannot be ruled out entirely and should be acknowledged as a potential limitation.

### 3.6 Data analysis

Data were analyzed using IBM SPSS Statistics (Version 25.0), AMOS (Version 23.0), and the SPSS PROCESS macro (Version 4.2; Hayes, 2017). Frequency analyses, reliability testing (McDonald’s *ω*), exploratory factor analysis (EFA), confirmatory factor analysis (CFA), and measurement model analyses were conducted to evaluate the psychometric properties of the instruments. To assess potential common method bias, a common method factor analysis was performed.

For the analysis of moderated mediation pattern, PROCESS Model 83 was applied, allowing for the testing of conditional indirect effects in the presence of moderators. Bootstrapping procedures with 5,000 resamples and a 95% bias-corrected confidence interval were employed to determine the significance of the effects. Additionally, independent and moderator variable were mean-centered to minimize potential multicollinearity in the moderation and moderated mediation analyses. The conditional effects and conditional indirect effects were examined at three levels of the moderator: one standard deviation below the mean (M − 1SD), the mean (M), and one standard deviation above the mean (M + 1SD).

## 4 Research results

### 4.1 Correlation between main variables

The results of the Pearson bivariate correlation analysis among the study variables are presented in Table 1. Hope, peer relationship, sports participation motivation, and sports engagement were all significantly and positively correlated with one another. In contrast, grit was significantly and negatively correlated with peer relationship, suggesting an inverse association between these two variables. In addition, the correlation coefficients among the independent variables were all below 0.70, suggesting that multicollinearity was not a concern in subsequent analyses (Tabachnick and Fidell, 2019).

**Table 1:** Correlation and descriptive statistics.

Categories	1	2	3	4	5
1. Grit	1				
2. Peer relationship	−0.263^**^	1			
3. Hope	0.393^**^	0.259^**^	1		
4. Sports participation motivation	0.310^**^	0.094^*^	0.474^**^	1	
5. Sports engagement	0.244^**^	0.186^**^	0.510^**^	0.669^**^	1
M	3.2957	3.6283	3.7775	4.1373	3.7262
SD	0.73647	0.66386	0.63866	1.32382	0.84767

^*^p < 0.05, ^**^p < 0.01.

In the descriptive statistical analysis, all variables demonstrated values above the median. Among them, sports participation motivation showed the highest mean score (M = 4.1373).

### 4.2 Moderated mediation pattern of peer relationship

**Figure 2:**
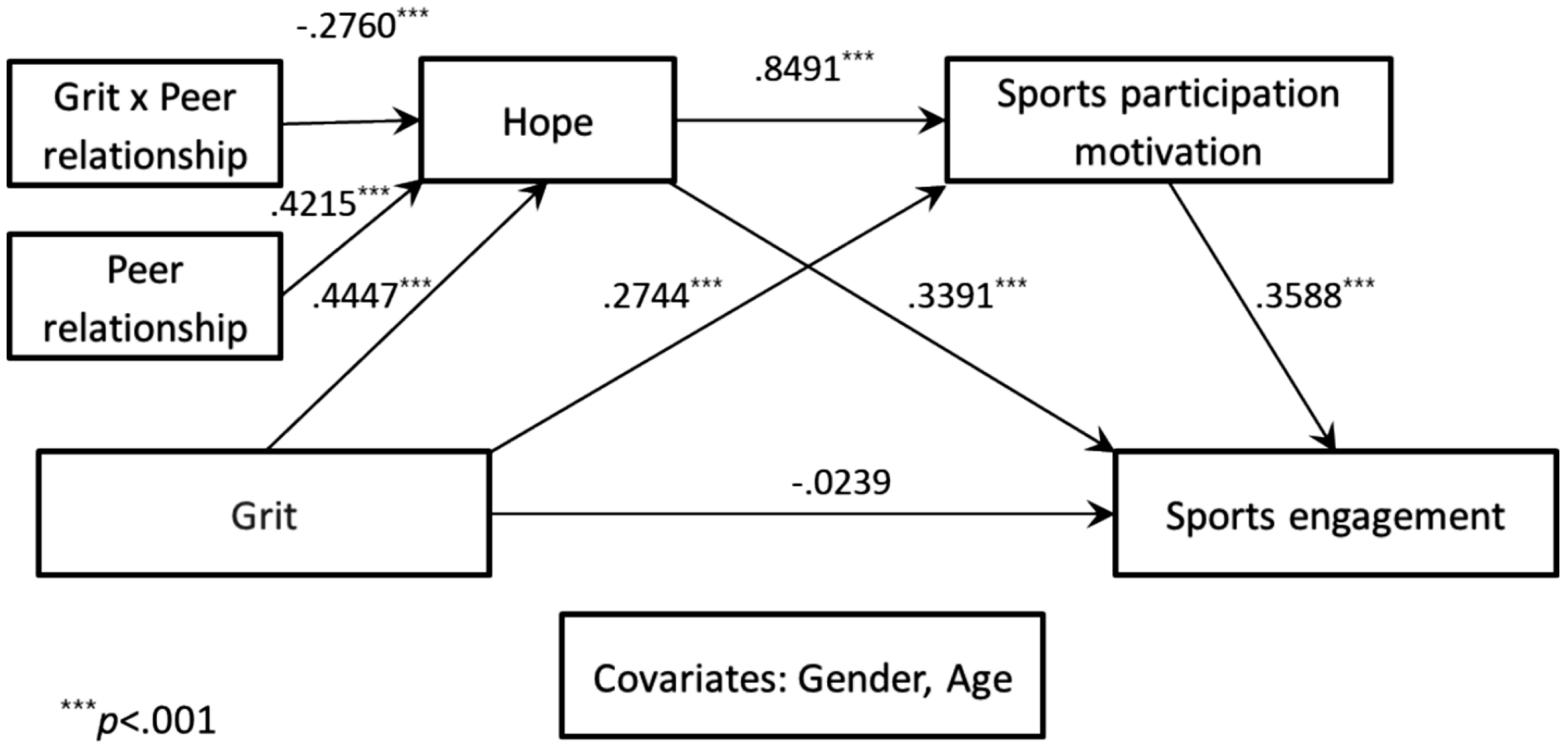
Statistical model of moderated mediation effect of peer relationship.

**Figure 3:**
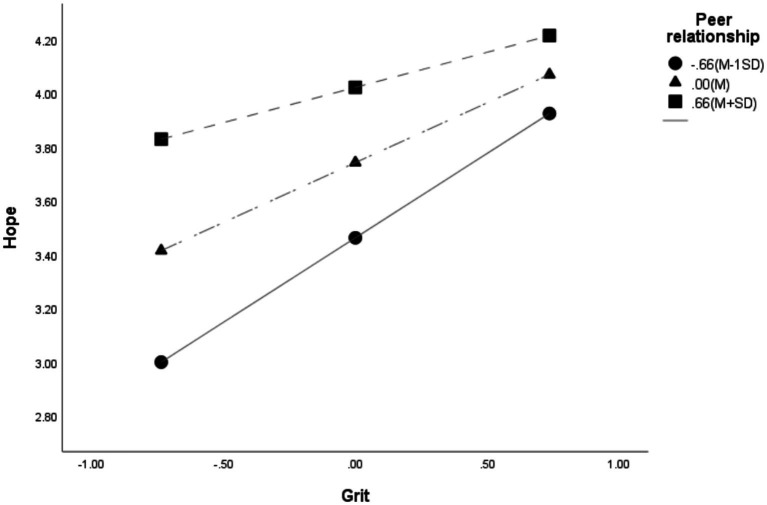
Moderating effect of peer relationship on the relationship between grit and hope.

To examine whether peer relationship moderates the serial mediation association of hope and sports participation motivation in the association between grit and sports engagement, the SPSS PROCESS macro (Model 83; Hayes, 2017) was employed. Grit and peer relationship were mean-centered prior to analysis, and gender and age were included as control variables. The results are presented in Figures 2, 3 and Tables 2, 3.

**Table 2:** Moderating effect of peer relationship in the moderated mediation model.

Classification		Mediating variable model (DV: hope)			Mediating variable model (DV: sports participation motivation)			Dependent variable model (DV: sports engagement)		
		Coeffect	SE	*t* value	Coeffect	SE	*t* value	Coeffect	SE	*t* value
Constant		4.3595	0.4751	9.1762^***^	2.7584	1.1513	2.3959^*^	0.8417	0.6049	1.3914
IV	Grit	0.4447	0.0325	13.6810^***^	0.2744	0.0782	3.5108^***^	−0.0239	0.0413	−0.5774
M1	Hope				0.8491	0.0877	9.6822^***^	0.3391	0.0499	6.7951^***^
M2	Sports participation motivation							0.3588	0.0233	15.4158^***^
Moderator	Peer relationship	0.4215	0.0372	11.3420^***^						
Interaction	Grit x peer relationship	−0.2760	0.0390	−7.0757^***^						
Higher order test	*R*^2^ change	0.0633								
	*F*	50.0661^***^								
Covariates	Gender	0.0840	0.0552	1.5235	0.1831	0.1222	1.4975	−0.1272	0.0640	−1.9877
	Age	−0.0358	0.0255	−1.4016	−0.1034	0.0574	−1.8033	0.0112	0.0301	0.3714
Model Summary	*R* ^2^	0.3640	0.2488	0.5009						
	*F*	57.5650^***^	42.7281^***^	100.9599^***^						

Mediating variable model (hope)					
Conditional effects of the grit at values of the hope					
Peer relationship	Effect	se	*t* value	LLCI^*^	ULCI^**^
−0.6639 (M-1SD)	0.6280	0.0426	14.7267^***^	0.5442	0.7118
0.0000 (M)	0.4447	0.0325	13.6810^***^	0.3809	0.5086
0.6639 (M + 1SD)	0.2615	0.0405	6.4639^***^	0.1820	0.3410

Moderator value(s) defining Johnson–Neyman significance region(s)		
Value	% below	% above
1.2130	96.2672	3.7328

Conditional effect of focal predictor at values of the moderator					
Peer relationship	Effect (B)	se	*t* value	LLCI^*^	ULCI^**^
−2.0033	0.9977	0.0862	11.5679^***^	0.8283	1.1672
1.2029	0.1127	0.0556	2.0258^*^	0.0034	0.2220
1.2130	0.1099	0.0559	1.9647	0.0000	0.2198
1.3717	0.0661	0.0611	1.0821	−0.0539	0.1861

^*^p < 0.05, ^**^p < 0.01, ^***^p < 0.001.

*LLCI=Bootstrap lower bound within 95% confidence interval.

**ULCI=Bootstrap upper bound within 95% confidence interval.

**Table 3:** Analysis of direct effect and conditional indirect effect.

Direct effect (grit → sports engagement)				
Effect	se	*t* value	LLCI^*^	ULCI^**^
−0.0239	0.0413	−0.5774	−0.1051	0.0573

Conditional indirect effects of X on Y				
Grit → hope → sports participation motivation → sports engagement				
Peer relationship	Effect	BootSE	BootLLCI	BootULCI
−0.6639 (M-1SD)	0.1913	0.0382	0.1206	0.2716
0.0000 (M)	0.1355	0.0263	0.0871	0.1908
0.6639 (M + 1SD)	0.0797	0.0183	0.0466	0.1180

Index of moderated mediation				
Peer relationship	Index	BootSE	BootLLCI	BootULCI
	−0.0841	0.0215	−0.1308	−0.0465

^***^p < 0.001.

^*^LLCI = Lower limit within the 95% confidence interval of the bootstrapped indirect effect.

^**^ULCI = Upper limit within the 95% confidence interval of the bootstrapped indirect effect.

Grit exerted a significant positive effect on hope (B = 0.4447, *p* < 0.001). In turn, hope (M2) significantly predicted sports participation motivation (M2) (B = 0.8491, *p* < 0.001), which subsequently had a significant positive effect on sports engagement (B = 0.3588, *p* < 0.001). However, grit did not have a significant direct effect on sports engagement (B = −0.0239, *p* > 0.05). These findings indicate that hope and sports participation motivation fully and sequentially mediated the relationship between grit and sports engagement.

The interaction between grit and peer relationship significantly predicted hope (B = −0.2760, *p* < 0.001), demonstrating that peer relationship moderated the effect of grit on hope by attenuating its positive influence. The change in explained variance was statistically significant (Δ*R*^2^ = 0.0633, *F* = 50.5650, *p* < 0.001), indicating that the inclusion of the interaction term meaningfully improved the model.

Conditional effects analysis further confirmed this moderating pattern. Although the conditional effects of grit on hope were significant at all three tested levels of the moderator, the magnitude of the effect decreased as peer relationship increased from M − 1 SD to M + 1 SD. This pattern suggests that peer relationship functions as a suppressor, weakening the positive association between grit and hope.

The Johnson–Neyman technique was applied to identify the region of significance for the conditional effect. The effect of grit on hope remained significant when peer relationship values were below 1.2130, a range that included 96.3% of the participants. Conversely, when peer relationship values were 1.2130 or higher, the conditional effect was not significant, representing 3.7% of the sample.

The significant conditional effect is illustrated in Figure 3. Overall, hope increased as grit increased; however, the slope was steeper for individuals with low levels of peer relationship, whereas the increase was more gradual for those with high levels of peer relationship.

Next, to examine the moderated mediation effect of grit on sports engagement, both direct and conditional indirect effects were analyzed. The direct effect of grit on sports engagement was not significant (B = −0.0239, 95% CI [−0.1051, 0.0573], *p* > 0.05).

Subsequently, the conditional indirect effect of peer relationship was tested in the pathway from grit to sports engagement through hope and sports participation motivation. The conditional indirect effects were significant at all three levels of the moderator (M − 1 SD, M, and M + 1 SD), indicating that the indirect effect of grit on sports engagement varied depending on the level of peer relationship. Moreover, as peer relationship increased from M − 1 SD to M + 1 SD, the magnitude of the conditional indirect effect decreased, demonstrating a weakening pattern.

The index of moderated mediation for peer relationship was B = −0.0841, and the 95% bootstrap confidence interval did not include zero (−0.1308 to −0.0465), confirming that the conditional indirect effect was statistically significant.

## 5 Discussion and conclusion

The present study found that grit, hope, sports participation motivation, and sports engagement were positively associated, suggesting that psychological strengths and sport-related behaviors may be interrelated in ways that mutually reinforce one another. This pattern is consistent with prior findings showing that grit and hope predict athletes’ motivation and adaptive functioning (Longakit et al., 2025; Nothnagle and Knoester, 2025), and contribute to well-being and health-promoting behaviors (Lee and Oh, 2025; Song et al., 2025).

From a theoretical standpoint, these relationships may be understood through self-determination theory and hope theory. Grit reflects sustained effort over time, which fosters goal-directed thinking—a central element of hope. Hope, in turn, activates motivational pathways by enhancing perceived agency and pathway thinking, leading to greater investment in sports-related activities. These mechanisms suggest a sequential cognitive–motivational process through which perseverance is translated into engagement via hope and intrinsic motivation.

Although the peer relationship scale assessed positive aspects of peer interactions, its unexpected negative correlation with key outcomes may reflect the complex influence of peer salience in competitive sport contexts. In such environments, even supportive peer bonds can increase sensitivity to evaluative standards and provoke self-comparison, which may reduce intrinsic motivation and persistence (Smith et al., 2006). This dynamic can be especially pronounced in collectivist cultures, where maintaining group harmony and avoiding underperformance relative to peers carry high social value (Markus and Kitayama, 1991).

Alternatively, the attenuating effect of peer relationships may stem not from peer support itself, but from the pressure that arises when peer expectations are internalized as performance demands. This highlights the possibility that what appears as social support may simultaneously function as a source of evaluative stress. Further, unmeasured factors such as peer competitiveness or status anxiety may account for the observed suppression of hope in high peer-salience conditions.

Boundary conditions should also be acknowledged. The findings may be culturally bounded, particularly in East Asian university contexts where group-based evaluations and collective responsibility are emphasized. Moreover, the peer effect observed here may be limited to structured or graded sport participation, as opposed to voluntary, informal, or elite sport settings. Age, gender, or prior athletic experience may further moderate the observed pathways.

A key contribution of this study is the identification of a significant moderated mediation pathway, in which peer relationships conditionally influence the indirect effect of grit on sports engagement through hope and sports participation motivation. This conditional indirect effect illustrates that the psychological impact of grit is not uniform, but instead depends on the social context—particularly the level of peer support perceived by the individual. Such findings extend beyond traditional mediation models by demonstrating how and when grit operates through internal resources like hope and motivation. Specifically, grit predicted higher hope only under conditions of lower peer support, suggesting that grit may serve as a compensatory strength in less supportive social climates. In contrast, when peer support is high, individuals may rely less on their internal perseverance, thus attenuating grit’s impact. This nuanced understanding underscores the theoretical importance of social moderators in motivational models of sport behavior and offers practical implications for tailoring interventions according to peer dynamics.

The interpretation of peer relationships has been revised to reflect the positive directional scoring of the scale, where higher scores indicate greater peer support. In this context, the moderating effect should not be attributed to dysfunctional or negative peer environments, but rather to the specific interaction between peer relationships and grit. Although peer relationships appear to be generally positive, the negative interaction observed with grit may be understood through the unique nature of grit itself—defined by long-term perseverance and consistency of interest. Within the university setting, maintaining strong peer relationships may sometimes temporarily disrupt or dilute the sustained personal focus required for grit, particularly when social expectations or group-oriented activities demand short-term flexibility or relational harmony. This does not imply that peer relationship is harmful, but rather that in highly interconnected peer environments, individuals may be less reliant on their internal perseverance, as social cohesion or peer obligations may unintentionally interfere with the stable pursuit of long-term personal goals.

This study contributes to the growing literature by highlighting how the psychological function of grit is embedded within a broader social-motivational system. While much prior research has positioned grit as a universally positive predictor, our findings suggest that its efficacy depends on intermediary motivational constructs and the surrounding peer climate. This nuance aligns with emerging integrative models that emphasize the interaction between individual dispositions and contextual supports in predicting engagement (Credé et al., 2017; Walton and Yeager, 2020).

Notably, grit did not exert a significant direct effect on sports engagement in the conditional process model, despite showing a positive bivariate correlation. This pattern suggests that grit alone may be insufficient to drive sustained engagement without the presence of supportive psychological mechanisms. Specifically, grit’s impact appears to operate indirectly through constructs such as hope and participation motivation, which themselves are shaped by the quality of peer relationships. This finding aligns with emerging perspectives suggesting that grit may require a motivationally supportive environment to translate into action, particularly in domains such as sport that involve social interaction, evaluative pressures, and goal regulation. The results therefore emphasize the importance of cognitive-affective mediators and social context in activating the behavioral potential of grit, rather than treating grit as a uniformly autonomous predictor of engagement.

These findings collectively highlight the dynamic interplay between personal strengths and social environments in shaping behavioral outcomes in sport, and emphasize the need for future research to adopt integrative, context-sensitive models of motivation.

This study has several limitations that should be acknowledged. First, the cross-sectional design restricts the ability to draw causal inferences regarding the relationships among grit, hope, peer relationship, sports participation motivation, and sports engagement. Second, peer relationship was assessed as a broad, undifferentiated construct, and the study did not distinguish among specific dimensions such as peer relationship, conflict, or exclusion, which may have distinct effects on psychological and behavioral outcomes. Third, the sample consisted exclusively of sports participants, limiting the generalizability of the findings to athletes in other sports or to different age groups. Fourth, to align with theoretical models, several multidimensional constructs—such as grit, hope, sports participation motivation and peer relationships—were modeled as unidimensional despite theoretical and empirical indications of multi-factor structures. While this approach was empirically driven, it may have limited construct precision by oversimplifying the complexity of these psychological constructs. Fifth, another notable limitation of this study is the pronounced gender imbalance within the sample, with over 70% of participants identifying as male. Given that previous research has identified gender-based differences in peer dynamics, sport participation motivation, and engagement patterns, this imbalance may limit the generalizability of the findings and introduce potential gender-related biases in model interpretation. As such, caution is warranted when extrapolating the results to more gender-balanced or female-majority populations. Sixth, EFA and CFA were conducted on the same dataset due to sample size limitations. As such, the CFA results should be interpreted with caution, and claims of confirmation are considered preliminary. Finally, the use of self-report measures introduces the possibility of social desirability bias and recall bias, which may have influenced participants’ responses.

Future research should address these limitations in several ways. Future research should address these limitations in several ways. First, longitudinal or experimental designs are needed to clarify the causal pathways linking grit, hope, peer relationship, and sport participation motivation. Second, future studies should differentiate between supportive and conflictual peer subdimensions to better understand their unique and potentially divergent effects. Third, expanding research to include athletes from various sports and developmental stages would enhance the generalizability of the findings. Future studies are encouraged to adopt more rigorous and stratified sampling strategies to ensure a more demographically representative participant pool. Fourth, future studies consider alternative modeling strategies, such as second-order CFA or dimension-level analyses, to more fully capture the multidimensional nature of these variables. Fifth, subgroup analyses based on gender should be conducted to determine whether the proposed model operates similarly across male and female participants. This approach would enhance the external validity of the findings and contribute to a more nuanced understanding of gender-specific mechanisms in sport-related psychological constructs. Sixth, future studies are encouraged to employ a split-sample approach or utilize independent samples to separately conduct exploratory and confirmatory factor analyses. This would allow for more robust validation of the factor structure and reduce the risk of overfitting. Finally, further research should focus on the procedural strategies adopted in the research design, as these likely play a more critical role in mitigating method bias.

## Data Availability

The raw data supporting the conclusions of this article will be made available by the authors, without undue reservation.
